# Timing of *embryonic* quiescence determines viability of embryos from the calanoid copepod, *Acartia tonsa* (Dana)

**DOI:** 10.1371/journal.pone.0193727

**Published:** 2018-03-07

**Authors:** Birgitte Nilsson, Benni Winding Hansen

**Affiliations:** Department of Science and Environment, Roskilde University. Universitetsvej 1, Roskilde, Denmark; Stazione Zoologica Anton Dohrn, ITALY

## Abstract

Like 41 other calanoid copepods, *Acartia tonsa*, are capable of inducing embryonic quiescence when experiencing unfavorable environmental conditions. The ecdysone-signaling cascade is known to have a key function in developmental processes like embryogenesis and molting of arthropods, including copepods. We examined the role of *ecdysteroid-phosphate phosphatase* (*EPPase*), *ecdysone receptor* (*EcR*), *ß fushi tarazu transcription factor 1* (*ßFTZ-F1*), and the *ecdysteroid-regulated early gene E74* (*E74*), which represent different levels of the ecdysone-signaling cascade in our calanoid model organism. Progression of embryogenesis was monitored and hatching success determined to evaluate viability. Embryos that were induced quiescence before the gastrulation stage would stay in gastrulation during the rest of quiescence and exhibited a slower pace of hatching as compared to subitaneous embryos. In contrast, embryos developed further than gastrulation would stay in gastrulation or later stages during quiescence and showed a rapid pace in hatching after quiescence termination. Expression patterns suggested two peaks of the biological active ecdysteroids, 20-hydroxyecdysone (20E). The first peak of 20E was expressed in concert with the beginning of embryogenesis originating from yolk-conjugated ecdysteroids, based on *EPPase* expression. The second peak is suggested to originate from *de novo* synthesized 20E around the limb bud stage. During quiescence, the expression patterns of *EPPase*, *EcR*, *ßFTZ-F1*, and *E74* were either decreasing or not changing over time. This suggests that the ecdysone-signaling pathway play a key role in the subitaneous development of *A*. *tonsa* embryogenesis, but not during quiescence. The observation is of profound ecological and practical relevance for the dynamics of egg banks.

## Introduction

Copepods are of high ecological importance by linking energy and matter from phytoplankton to higher trophic levels of the pelagic marine food web [1]. The calanoid copepod, *Acartia tonsa*, inhabits estuaries and nearshore environments of temperate waters [2]. Besides a high tolerance towards environmental changes, *A*. *tonsa* are like 41 other calanoids capable of coping with seasonality and stressful conditions by producing dormant eggs where embryogenesis is on a halt [3]. Subitaneous eggs, hatch within a few days after oviposition when the surrounding conditions are optimal. When conditions are sub-optimal, the embryos can survive by undergoing dormancy, which comprises following three types: quiescence, which is defined as retarded development, oligo-pause which is delayed hatching embryos and diapause, where development is arrested [3].

The dormant eggs sink to the bottom where they are buried and accumulate in the sediment egg bank. Depending on the type of dormancy and sediment conditions, the embryos can survive for years. When the sediment is being disturbed and dormant eggs return to the water column, where they under favorable conditions will hatch and recruit new copepods to the pelagic population [4,5].

Even though copepods are ecological important not much is known about the underlying embryonic mechanisms during subitaneous development and quiescence. Most studies are dealing with the post-embryonic development of calanoid copepods, but only a few studies concern the embryonic development in further details [6,7].

**Fig 1:**
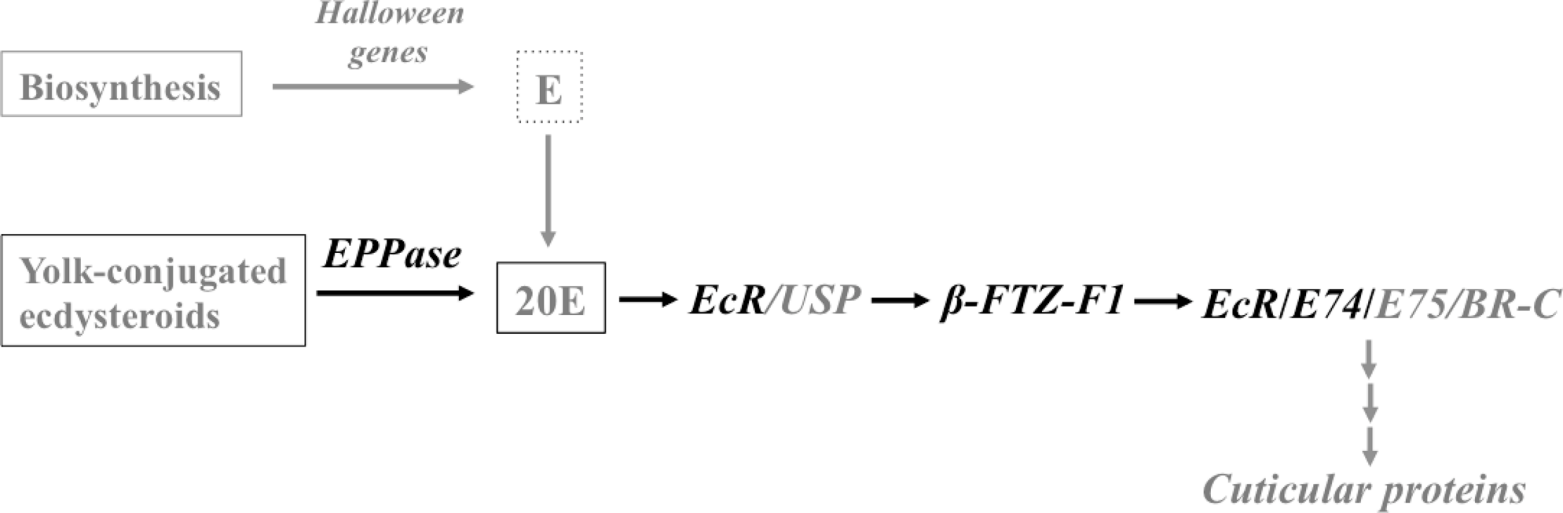
Simplified diagram of the ecdysone-signaling pathway. Yolk-conjugated ecdysteroids are released in free form by the action of ecdysteroid-phosphate phosphatase (EPPase) leading to the biological active 20-hydroxyecdysone (20E). High titers of 20E activate the Ecdysone Receptor (EcR) / Ultraspiracle (USP) complex, which targets down-stream stimulation of ß-fushi tarazu transcription factor 1 (ßFTZ-F1). ßFTZ-F1 stimulates the expression of ecdysteroids-responsive genes like early genes 74 and 75 (E74, E75) and the Broad-Complex (BR-C). The ecdysteroids-responsive genes stimulate the expression of cuticular protein genes, which ultimately will result in embryonic molting. Another possible route of the embryonic ecdysteroid-signaling pathway is the biosynthesis of ecdysone (E) by the Halloween genes, which is the pre-cursor of 20E. Genes used as representatives in gene expression analysis of the present study are marked with bold, black font.

Ecdysteroids is a group of polyhydroxylated sterols that in arthropods mediates embryonic development, molting, metamorphosis, and adult development by stimulating curticular protein (CP) production [8,9]. Embryonic ecdysteroids are secreted from ovarian follicle cells and converted into conjugates with yolk proteins [10–14]. During embryogenesis, the enzyme, ecdysteroid-phosphate phosphatase (EPPase), hydrolyzes conjugated ecdysteroids into free-form as the yolk-proteins continuously degrade [15,16] (Fig 1). In addition, embryonic ecdysteroids are also suggested to be *de novo* synthesized enzymes encoded by a set of genes called the Halloween genes [17] (Fig 1).

The biological active ecdysteroid, 20-hydroxyecdysone (20E) will upon interaction with a heterodimer receptor complex consisting of the ecdysone receptor (EcR) and ultraspiracle (USP), initiate a cascade of ecdysteroid responsive genes that ultimately will stimulate embryonic molting by targeting the expression of CP genes [9,18–20] (Fig 1). Embryonic molting is the deposition of embryonic cuticles [21,22].

Following interaction between 20E and the EcR/USP complex, the expression of *ß-fushi tarazu (ftz) transcription factor 1* (*ßFTZ-F1*) will be stimulated, and target down-stream ecdysone responsive genes, including the *early genes 74* and *75* (*E74*, *E75*) and the *Broad-Complex* [9]. Expression of *ßFTZ-F1* is timely restricted during development and have an important role in late embryogenesis and later molting processes by regulating the ecdysteroid-responsive genes, including E74 [9,23].

The aim of the present study is to examine subitaneous development and quiescence of copepod embryos using the model species, *A*. *tonsa*. The embryogenesis was monitored by visualization of DAPI-stained eggs during subitaneous development and quiescence. Hatching success was estimated to determine viability and gene expression of *EPPase*, *EcR*, *ßFTZ-F1*, and *E74* were analyzed since they represent four different levels of the ecdysone-signaling cascade leading to embryonic molting and development.

## Materials and methods

### Cultures

The *A*. *tonsa* strain (DFH-ATI) used for cultivation originated from Øresund (N 56°/E 12°; Denmark) and was isolated in 1981 [24]. The strain has for 30 years been maintained under constant salinity, temperature and light conditions (salinity 32, 17°C, dim light) and fed on a diet consisting of the mono-algae, *Rhodomonas salina* (identity code: K-1487). Three copepod cultures were set up prior the experiments in order to provide statistical replicates. The cultures were kept in 60 L flat-bottomed polyethylene tanks at the same stable conditions as mentioned above in 40 L 0.2μm-filtered seawater with gentle aeration. The copepods were fed *R*. *salina ad libitum* every day (>800 μg C L^-1^), which was cultivated in 2.0 L round-bottom glass flasks with F2 media [25,26]. Cultivation took place under stable conditions at 17°C with CO_2_ supply and light (PAR~80μE m^-2^ s^-1^). All experiments were conducted with 0.2μm-filtered seawater with a salinity of 32.

### Hatching success

**Fig 2:**
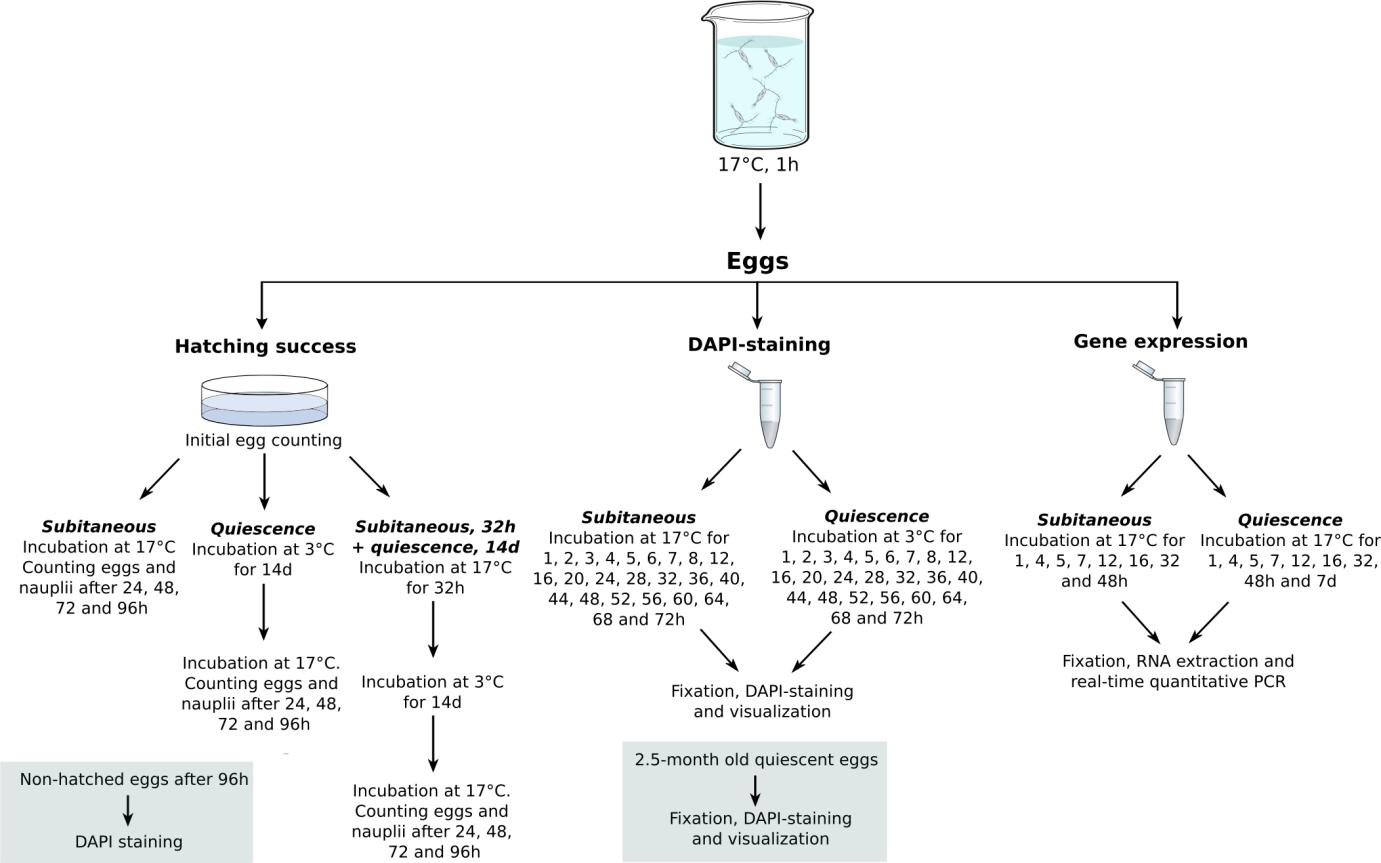
Experimental setup for determining hatching success, DAPI-staining and gene expression analysis of *Acartia tonsa* eggs. See text for details.

Adult copepods isolated with a 400μm mesh was transferred to 1.0 L beaker glasses containing seawater in order to spawn. After 1 h of incubation, the adults were separated from the eggs using a 125μm mesh on top of a 54μm mesh. Eggs were transferred to 50 mm in diameter petri dishes and incubated at 16.9 ± 0.1°C (mean ± SD) for subitaneous development of the embryos in 10 mL seawater. Eggs and nauplii were counted after 24, 48, 72 and 96 h in order to determine the hatching success (Fig 2). Subitaneous hatching success was performed with 6 replicates (624 ± 113 eggs per replicate, mean ± SD).

Based on the following DAPI visualization of embryogenesis, eggs were within 1 h after oviposition (n = 160 ± 24, 5 replicates) transferred to petri-dishes and incubated at 3.0 ± 0.2°C for 14 days to induce quiescence (Fig 2). Furthermore, additional embryos (n = 191 ± 26) were undergoing subitaneous development for 32 h at 16.9 ± 0.1°C before being incubated at 3.0 ± 0.2°C for 14 days for quiescence (Fig 2). Eggs and nauplii were following quiescence counted after 24, 48, 72 and 96 h to determine hatching success (Fig 2). The eggs that remained un-hatched after 96 h were selected and stained as described in the DAPI staining.

### DAPI staining

Adult copepods from the three individual cultures were isolated with a 400μm mesh and transferred to 1.0 L beaker glasses containing 800 mL seawater with gentle aeration. The copepods were fed *R*. *salina* and incubated to spawn for 1 h in darkness at 16.9 ± 0.1°C (mean ± SD). The adults, and other life-stages were separated from the eggs by using a 125μm mesh on top of a 54μm mesh to collect the eggs. Eggs were transferred to 1.5 mL Eppendorf tubes containing seawater and incubated at 16.9 ± 0.1°C. Sampling was done each hour from 1 to 8 h, and for every fourth hours from 8 h to 72 h (Fig 2). The samples were centrifuged at 10.000 rpm for 30 seconds in order to pellet the eggs. Residual seawater was removed by pipetting and eggs were fixed by sodium tetraborate-buffered 4% formaldehyde in seawater, in order to stop the biochemical activities in the embryos and increase mechanical strength [27]. The seawater used for dilution of the formaldehyde was the same as used for cultivation of the copepods in order to avoid the undesired effects of osmotic stress in the embryos. Eggs treated with the fixation solution were stored at 3 ± 0.2°C (mean ± SD) in darkness for a minimum of 24 h before further processing.

Embryos, from each developmental time-step, were stained with DAPI according to [28]. De-ionized water was used to cause osmotic stress to such a degree that it allowed the uptake of the fluorescent molecule into the embryo and the nuclei. Ethanol-rinsed eggs from each sample were transferred to a depression slide (n = 20) containing 100 μL of 10 μg/mL DAPI and then covered with a coverslip. The depression slides containing the treated eggs were incubated at 3 ± 0.2°C (mean ± SD) in darkness for 24 h.

Besides the progressive development during subitaneous and quiescent state, the non-hatching eggs described under hatching success, as well as ~2.5-months old cold-stored eggs, were also DAPI-stained as described here. The ~2.5-months old cold-stored eggs originated from the *A*. *tonsa* egg-bank at Roskilde University harvested July the 25^th^, 2017 (stained October the 3^rd^, 2017).

The subitaneous eggs were examined with a Nikon Eclipse Ti-U inverted microscope (Nikon, Nikon Instruments Europe B.V.) equipped with a 60X dry Plan Apo objective. The epi-illumination was made by a LED epifluorescence UV (365 nm) light source (CoolLED ltd, Andover, UK), using Nikon´s UV-2B filter set (excitation at 330–380; emission at >435nm). Images were taken by a DS-QiMc camera (Nikon, Nikon Instruments Europe B.V.) powered by NIS elements™ software package (Nikon, Nikon Instruments Europe B.V). Images were made of multiple focus layers to create full depth of field images by scanning images through the embryos. After assembly of multiple focus layers, the final image was de-convoluted using Photiosity (version 1.7.0, Rolf Geprägs, Hamburg, Germany) with a grid size of 25 pixels and 100% noise control.

Quiescent eggs were examined with an Axio Vert.A1 FL inverted microscope with an LD EC Epiplan-Neofluar 50x/0.55 HD DIC M27 objective (Carl Zeiss, Welwyn Garden City, UK). Epi-illumination was provided by a LED epifluorescent UV (365 nm) HXP 120 C light source, using a DAPI filter (Carl Zeiss, Welwyn Garden City, UK). Images of the embryos were taken using an AxioCam MRc digital camera and the AxioVision (ver. 4.8) analysis software by automatic Z-stack scanning the embryos through in 20 slides of 2μm each (Carl Zeiss, Welwyn Garden City, UK). Convolution of the assembled images where processed in the same way as for the subitaneous embryos.

### Gene expression analysis

Based on the visualization of the embryonic development with DAPI-staining embryos were sampled for real-time quantitative PCR (qPCR) according to the developmental times given in S1 Table. For each of the developmental-times quadruplicate samples, each containing 100 eggs, was collected, flash frozen and stored at -80°C until further processing (Fig 2).

Total RNA was extracted using the RNeasy Mini Kit (Qiagen GmbH, Hilden, Germany). To ensure complete homogenization, embryos were first homogenized in 50 μL RLT buffer using disposable micro-pestles, after which additional 250 μL RLT buffer was added. The samples were vortexed for 5 sec and then processed according to the manufacturer’s protocol, with a final elution volume of 30 μL in RNase-free water. RNA concentration and purity were measured using a Nano-Drop 2000/2000c spectrophotometer (ThermoFisher Scientific Inc., USA). RNA integrity was tested on a denatured 1% agarose gel stained with ethidium bromide. Only samples with 280/260 absorbance’s between 1.8 and 2.0—and two distinct bands, corresponding to 28s and 18s rRNA, on the gel were used for further analysis.

An aliquot of 50 ng RNA from each sample was transcribed into cDNA with the QuantiTect Reverse Transcription Kit (Qiagen GmbH, Hilden, Germany) according to manufacturers’ protocol with the gDNA wipe-out treatment for removal of genomic DNA. The qPCR primers that are listed in S2 Table were produced in Geneious® (ver. 9.1.6, Biomatters Ltd, New Zealand). Sequences of *ecdysteroid-regulated early gene E74* (*E74*), *ecdysone receptor* (*EcR*), *ecdysteroid-phosphate phosphatase* (*EPPase*), *ß fushi tarazu transcription factor 1* (*ßFTZ-F1*), *Histone 3* (*HIST*) and *ATP synthase* (*ATPS*) from arthropod species found in the NCBI database were used to BLAST against the *A*. *tonsa de novo* transcriptome (accession no. GFWY00000000). Transcripts with the best matches with the used arthropod genes, in terms of similarity and e-values, were extracted from the transcriptome and used to generate the primers (S2 Table). The two reference genes, *HIST* and *ATPS*, were prior the study selected among 7 different genes to be the most stable across different developmental stages (eggs, adults) and stressors (salinity shock, handling—and density stress) after assessment with the geNorm method in the NormqPCR R package [29].

To ensure the identity of the primer product sequences originated from the *A*. *tonsa* transcriptome, conventional PCR were used to generate PCR products and submitted for sequencing at Eurofins Genomics (Ebersberg, Germany).

The Brilliant® II Master Mix (Sigma-Aldrich, USA) kit was used for setting up qPCR according to manufactures protocol using 2 μL cDNA as template. The reactions were run on Stratagene Mx3005P (AH Diagnostics, Aarhus V, Denmark) thermal cycler as follows: [95°C/15 min]; 40 cycles: [95°C/30s] [58°C/60s] [72°C/30s]. At the end of the cycling program, a melting curve analysis where added. Each replicate sample was run in technical triplicates. PCR amplification of each primer was prior the gene expression analysis determined by the standard curve method, and all had efficiencies above 80%. Gene expression was normalized using the geometric mean of the two reference genes *HIST* and *ATPs* and the 2^ΔΔ-CT^ method to estimate relative mRNA levels [30].

### Statistics

Since the gene expression levels were heavily skewed on the linear scale, the relative mRNA levels were log2 transformed prior the statistical analysis. Statistical significance of developmental time versus log2 fold change (log2FC) was determined using a one-way ANOVA followed by a Turkey’s post-hoc test. All data were tested for normality, homogeneity, and independence prior the ANOVA analysis.

Since count-data (e.g. hatching percentage are based on counts) inheritable are non-normally distributed a permutation analysis of variance (PERMANOVA) was conducted with 10,000 permutations. Because the interaction effect was significant we used a permutation t-test to compare hatching success means of the four time-points (i.e., 24, 48, 72, and 96 h) for the three treatments (i.e., subitaneous eggs, quiescent eggs and eggs undergoing subitaneous development for 32 h followed by quiescence). All data analysis and statistics were done using R [31].

## Results

### Hatching success

After 24 h of subitaneous development, 9.0 ± 2.9% (mean ± SD) the embryos had developed into fully hatched nauplii. The hatching success after 48 h, 72 h, and 96 h were 73.7 ± 2.9%, 87.2 ± 3.3%, and 89.6 ± 4.1%, respectively (Table 1). For embryos undergoing 14 d of quiescence initiated maximum 1 h after oviposition had after 24, 48, 72, and 96 h following hatching success: 0.6 ± 0.7%, 49.8 ± 3.6%, 63.9 ± 4.4%, and 82.7 ± 2.8% (Table 1). Embryos developed to the LB stage (32 h), followed by 14 d of quiescence, exhibited hatching success of 81.0 ± 2.9%, 86.9 ±1.9%, 87.8 ± 2.8%, and 89.7 ± 2.5% after 24, 48, 72, and 96 h, respectively (Table 1). The number of eggs was consistent with the resulting hatched nauplii.

**Table 1:** Hatching success (%) of *Acartia tonsa* eggs after subitaneous development, quiescence for 14 d, and subitaneous development for 32 h followed by 14 d of quiescence. Hatching success is given in percentage hatching (mean ± SD) after 24, 48, 72 and 96 h.

Development	24 h	48 h	72 h	96 h
Subitaneous development	9 ± 2.9%	73.7 ± 2.9%	87.2 ± 3.3%	89.6 ± 4.1%
Quiescence, 14 d	0.6 ± 0.7%	49.8 ± 3.6%	63.9 ± 4.4%	82.7±2.8%
Subitaneous development, 32 h Quiescence, 14 d	81.0 ± 2.9%	86.9 ±1.9%	87.8 ± 2.8%	89.7 ± 2.5%

The PERMANOVA indicated that both the main effects (i.e., time and treatments), as well the interaction between time and treatments were significant (p<0.05). All treatments were statistically significantly different after 24 and 48 h (p<0.05).

After 72h, the quiescent eggs had a significantly lower hatching success compared to the two other treatments (p<0.05). After 96h, the quiescent eggs were significantly different from eggs undergoing subitaneous development for 32 h followed by quiescence (p<0.05).

### DAPI staining

Embryogenesis was classified into 14 stages: 1 cell (S1), 2 (S2), 4 (S3), 8 (S4), 16 (S5), 32 (S6), (S7) and 128 cells to blastula (S8; in blastula, the cells are arranged in multiple rows), gastrulation (G), organogenesis (O), limb bud (LB), appendages appear (AA), early nauplii (EN) and final nauplii (FN). Only embryos with proper staining were used for stage determination.

**Fig 3:**
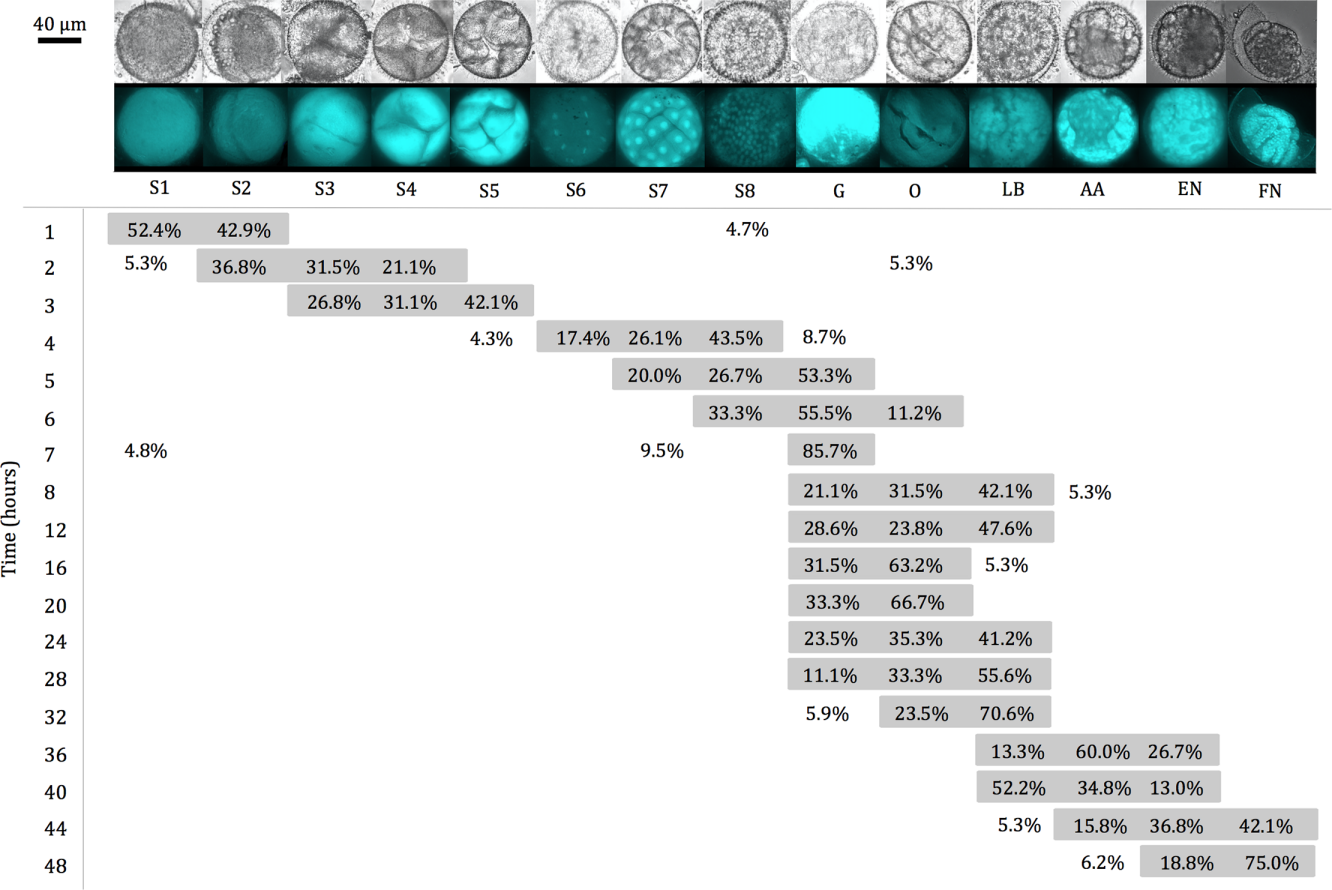
Percentages of embryonic stages during the subitaneous development of *Acartia tonsa*. On the top bright filter–and epifluorescent images can be seen for DAPI stained embryos of *A*. *tonsa*. Embryogenesis was divided into following stages: 1 cell (S1), 2 (S2), 4 (S3), 8 (S4), 16 (S5), 32 (S6), (S7) and 128 cells/blastula (S8), gastrulation (G), organogenesis (O), limb bud (LB), appendages appear (AA), early nauplii (EN) and final nauplii just before hatching (FN). For each of the developmental times, 26±2 embryos were used for stage estimation. Contrast and brightness were adjusted for publication.

**Fig 4:**
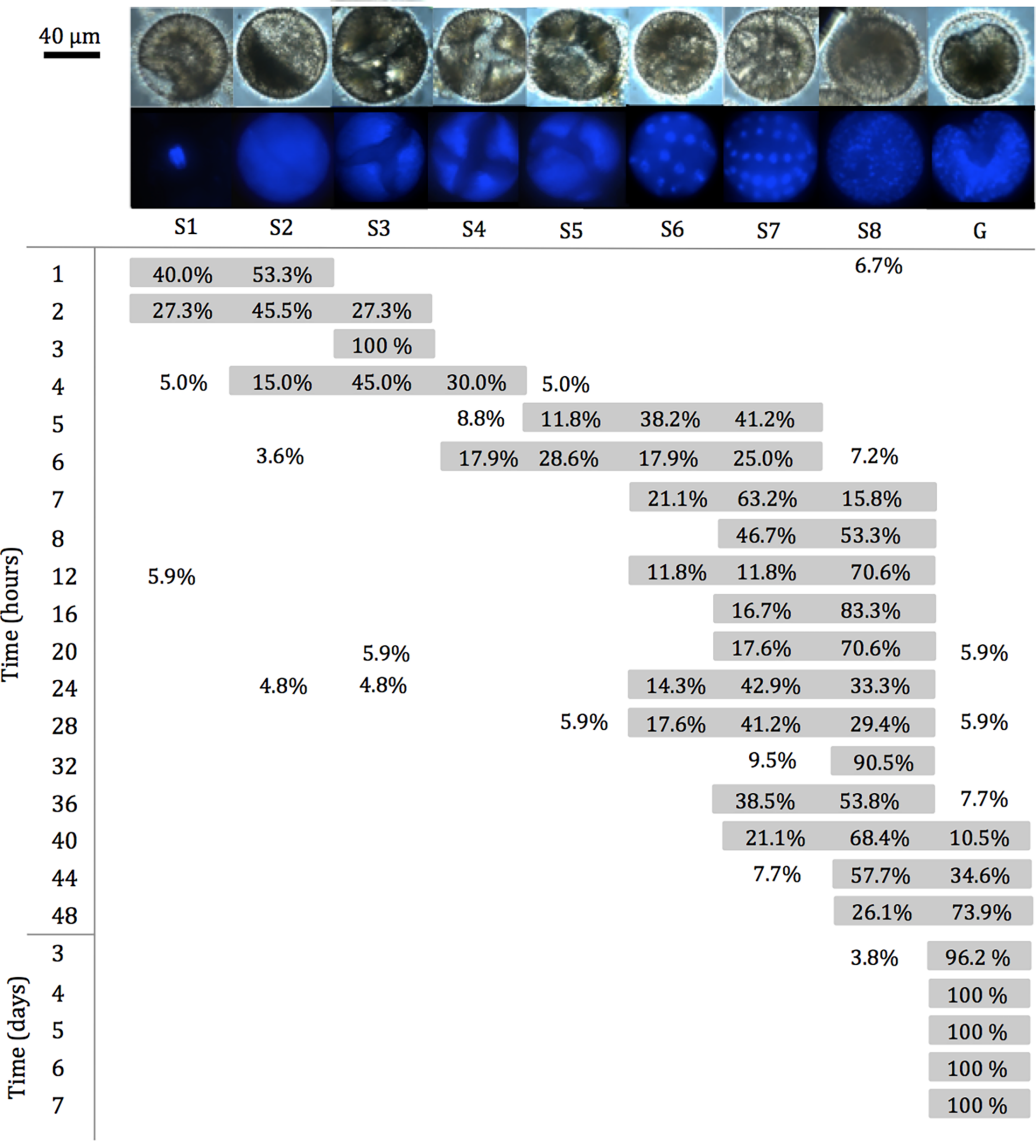
Percentages of embryonic stages during quiescence of *Acartia tonsa*. On the top bright filter–and epifluorescent images can be seen for DAPI stained embryos of *A*. *tonsa*. Embryogenesis was divided into following stages: 1 cell (S1), 2 (S2), 4 (S3), 8 (S4), 16 (S5), 32 (S6), (S7) and 128 cells/blastula (S8), gastrulation (G). For each of the developmental times, 19±4 embryos were used for stage estimation. Contrast and brightness were adjusted for publication.

During subitaneous development, the majority of embryos were present as S1 (52.4%, Fig 3) and S2 (42.9%, Fig 3) 1 h after oviposition. The brighter nucleus was faintly appearing approximately in the middle of the cell. The interference from the surrounding yolk made the visualization a bit unclear. Brighter areas observed in some of the embryos could indicate the polar bodies, but this was not studied further. The cleavage resulting in S2 was typically appearing asymmetric with the one cell larger than the second. From 1 to 4 h after oviposition, the embryogenesis gradually develop into S8 (majority 43.5%, Fig 3) by sub sequential cell cleavages. From 5 h after oviposition, the majority of the embryos start to enter G (Fig 3). The embryos were then present mainly in G, O, and LB until 32 h after oviposition (Fig 3). Hereafter the embryogenesis continued gradually until hatching around 48 h for most nauplii (Fig 3). Development during quiescence was slower than for subitaneous eggs (Fig 4). From 1 h (S1 and S2) to 36 h after oviposition following induced quiescence the embryos developed from S1 to S8 (Fig 4). Initial G was induced around 40 h, and the developmental progression stalled in G 48 h– 3 d after induced quiescence (Fig 4).

Embryos undergoing subitaneous development for 32 h followed by 14 d quiescence were also DAPI stained (n = 28) based on Figs 3 and 4. The staining exhibited that the embryos present were in following stages G (3.6%), O (7.1%), LB (32.1%), AA (21.4%), EN (21.4%) and FN (14.3%). As a control, eggs from the same batch, maximum 1 h after oviposition, had induced quiescence for 14 d followed by DAPI-staining (n = 19). All of the control embryos (100%) were in the G. Of the non-hatching eggs after 14 d of embryonic quiescence was 26.3% in G, 15.8% in O, 26.3% in LB, 21.1% in AA, and 5.6% of both EN and FN (n = 19). For non-hatching eggs with subitaneous embryonic development up till 32 h followed by 14 d of quiescence had following stage distribution; 25.0% in G, 16.7% in O, 25.0% in LB, 8.3% in AA, 16.7% EN, and 8.3% FN (n = 12). The ~2.5-month old cold-stored stained eggs (n = 31) the embryos were present in G (16.1%), O (12.9%), LB (22.6%), AA (19.4%), EN (25.8%), and FN (3.2%).

Only embryos that were properly stained were used for stage-determination. Some of the eggs exhibited proper DAPI staining only on the surface corresponding to bacteria and algae on the chorion. These were either dead or failed to be stained for some reason. Other eggs showed sign on punctuations and leakage. These embryos were all excluded from the study.

### Gene expression analysis

**Fig 5:**
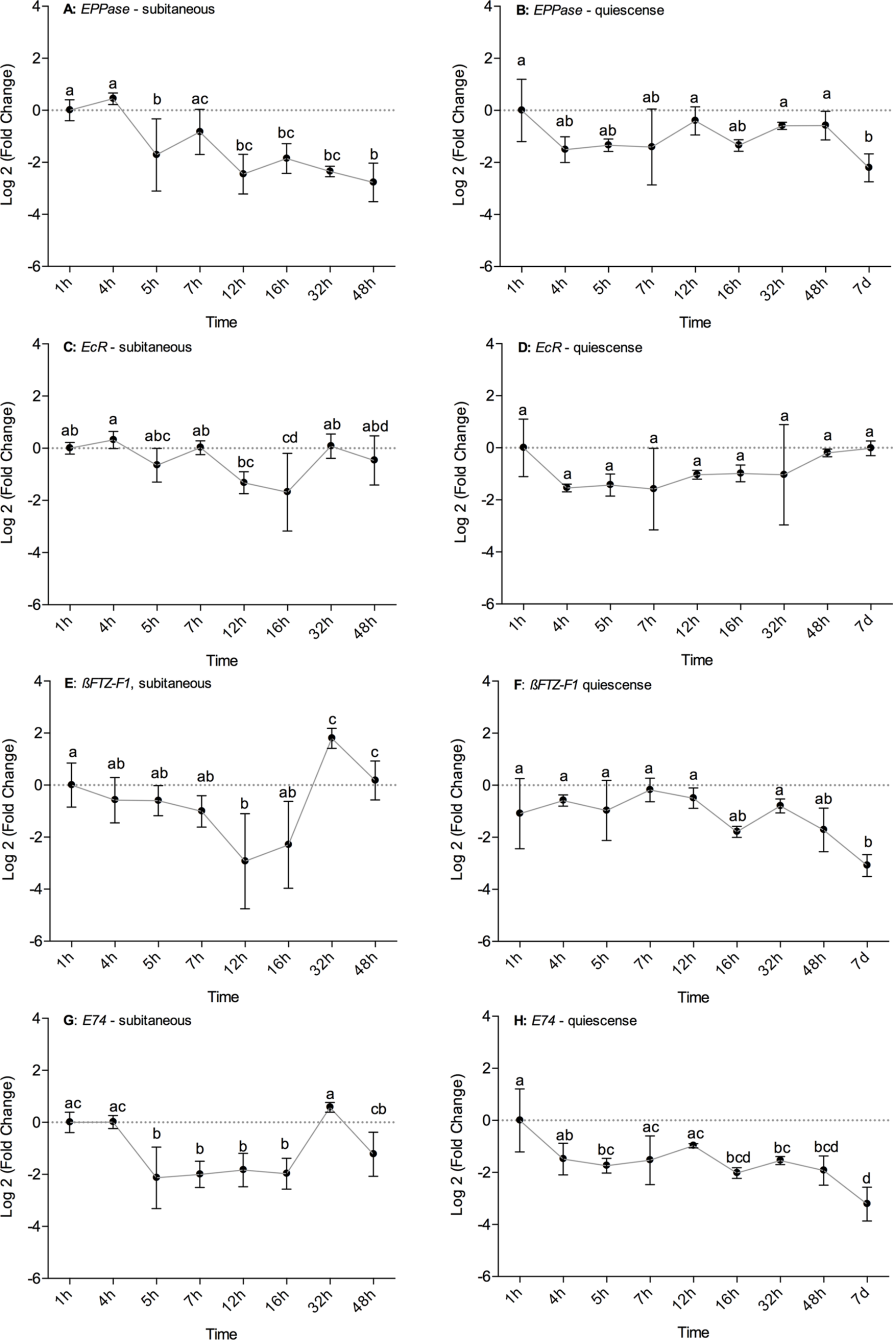
Real-time quantitative PCR analysis. A) *ecdysteroid-phosphate phosphatase (EPPas*e), subitaneous development; B) *EPPase*, quiescence; C) *ecdysone receptor* (*EcR*), subitaneous development; D) *EcR*, quiescence; E *ß fushi tarazu transcription factor 1* (*ßFTZ-F1*), subitaneous development; F) *ßFTZ-F1*, quiescence; G) *ecdysteroid-regulated early gene E74* (*E74*), subitaneous development; and H) E74, quiescence, of *Acartia tonsa*. For each of the selected developmental times, 4 biological replicates were collected. Each biological replicate contained 100 embryos of *A*. *tonsa*. The real-time quantitative PCR was performed with 3 technical replicates. Expression levels were normalized to the geometric mean of two stable reference genes, *histone 3* (*HIST*) and *ATP synthase* (*ATPS*). Error bars represent standard deviation. Tukey HSD was used to test for significant differences in gene expression (log2 FC) over time (h). Different letters indicate statistically significant differences (P < 0.05). Statistics were conducted using R and the graph was generated in GraphPad Prism (ver. 6).

A one-way ANOVA was carried out on gene expression (log2FC) over time (h) for *EPPase*, *EcR*, *ßFTZ-F1* and *E74* during subitaneous development and quiescence. The genes, *EPPase*, *ßFTZ-F1* and *E74*, had significant changes in gene expression over time for both the subitaneous and quiescent state (p < 0.05, Fig 5). *EcR* only exhibited significant change over time for subitaneous development (p < 0.05, Fig 5). Tukey’s HSD post hoc tests were carried out to estimate at which times gene expression differed significantly for the 4 genes.

The expression of *EPPase* at 1 and 4 h, during subitaneous development, both differed significantly from 5, 12, 16, 32 and 48 h (p < 0.05, Fig 5A). In addition, *EPPase* expression differed significantly after 48 h in comparison to 7 h (p < 0.05, Fig 5A). In the quiescent state, the gene expression of *EPPase* was significantly lower at 7 d than 1, 12, 32 and 48 h (p < 0.05, Fig 5B).

*EcR* did not exhibit any significant changes in gene expression over time during quiescence (Fig 5D). But 16 h of subitaneous development differed significantly from 1, 4, 7 and 32 h (p < 0.05, Fig 5C).

During the subitaneous embryonic development of *A*. *tonsa*, the expression of *ßFTZ-F1* was significantly different at 32 h in comparison to 4, 5, 7, 12 and 16 h (p < 0.05, Fig 5E). The 48 h did not differ from 32 h for *ßFTZ-F1* but were significantly different from 12 and 16 h (p < 0.05, Fig 5E). Furthermore, the 1 h of *ßFTZ-F1* differed significantly from 12 h (p < 0.05, Fig 5E). The expression of *ßFTZ-F1* was significantly lower after 7 d of quiescence in comparison to 1, 4, 5, 7, 12 and 32 h (p < 0.05, Fig 5F).

*E74* had a significant change in gene expression at 1 and 4 h in comparison to 5, 7, 12 and 16 h (p < 0.05, Fig 5G) during subitaneous embryonic development. Gene expression of *E74* at 32 h was significantly different from 5, 7, 12, 16 h, and 48 h (p < 0.05, Fig 5G). During quiescence, the expression of *E74* at 1 h was significantly higher in comparison to the other times, except 7 h (p < 0.05, Fig 5H). Furthermore, the expression of E74 was significantly lower at 7 d quiescence in comparison to 1, 4, 5, 7, 12 and 32 h (p < 0.05, Fig 5H).

## Discussion

To examine the role of ecdysteroids during subitaneous development and quiescence of *A*. *tonsa* embryos, we evaluated the egg hatching success, embryogenesis progression and gene expression of target genes involved in the ecdysone-signaling cascade.

Subitaneous eggs of *A*. *tonsa* had a hatching success of ~10% after 24 h, which increased to ~75% after 48 h and plateaued around ~90% after 48 and 96 h. Similar patterns of hatching success have been observed in studies using the same strain of *A*. *tonsa* and temperature as in the present study [32]. For instance, one study reported a hatching of 55% after 24 h and 90% after 48 h, while another reported hatching of ~25% after 24 h and ~65% after 48 h [32,33]. Regardless of the pattern leading to saturation of hatching succes, *A*. *tonsa* hatching reaches a plateau around 90% within 96 h [32–35].

Post-quiescent embryos exhibited a slower hatching rate in comparison to those with subitaneous development. Only about 1% of the embryos hatched within 24 h, that slowly increased to ~83% after 96 h. The post-quiescent hatching pattern is, however, similar to [33], that increased to ~84% after 96 h.

Compared to the DAPI visualization of subitaneous embryogenesis, the developmental progress stalled at the G stage around 48 h, were it remained for the rest of the quiescence. The slower hatching rate post-quiescence suggests that the embryos have to ‘wake up’ their development from their G stage before continuing subitaneous development, which takes longer time than if embryos were present in later stages than G (i.e. 32 h subitaneous embryos followed by 14 d quiescence).

To challenge this, embryos undergoing subitaneous development for 32 h, corresponding to the LB stage, were induced quiescence for 14 d. The hatching of these eggs was rapid, with a success ~82% after 24 h, which also plateaued around 90% after 96 h. The staining of these embryos revealed that only G–or developmental stages beyond that up to FN were present. The staining of ~2.5-month cold-stored eggs had similar embryonic stage-distribution. This suggests embryos that have not reached G will develop to this stage during quiescence and stay there, which also was confirmed by the positive control from the same egg batch (i.e. 14 d quiescence). If the embryos have progressed further than G during subitaneous development, the development will continue slowly towards FN, probably corresponding to the lower temperature conditions. These embryos will probably burn out of energy faster than embryos developing to and staying in G during quiescence.

For the present study, we examined embryos with an age of maximum ~2.5-months. Drillet et al. (2006) have shown that hatching of *A*. *tonsa* eggs was ~70–80% after 3 and 11-months, ~40% after 12-months, and no viable embryos after 20-months of cold-induced quiescence [36]. The duration limit in relation to quiescence is suggested to be due to energy-depletion, where fatty acid pools for embryonic development are reduced over time [37,38]. In the observed ~10% non-hatching eggs the embryos were all present in G or exceeding developmental stages. The development of these are probably at a slower pace in comparisons to other embryos from the same egg batch and can be categorized as delayed-hatching eggs according to Chen and Marcus (1997) [39].

The expression of *EPPase* at 1 and 4 h (S1-S8) differed from the rest of the subitaneous development, except at 7 h, which could be because of an outlier in the data. This suggests that *EPPase* have an important role in the beginning of *A*. *tonsa* embryogenesis. The expression after 1 and 4 h gradually decreased towards hatching. In the water-flee, *Daphnia magna*, *EPPase* expression have shown to be elevated during the first 6 hours of embryogenesis until the beginning of O [40]. This is comparable with the observed expression levels for *A*. *tonsa*, where *EPPase* is elevated just prior G. Gene knockout of *EPPase* have shown to result in the arrested development of *D*. *magna*, which confirms its essential role in early subitaneous development [40]. In the quiescent state, *EPPase* exhibited down-regulated expression after 1 h, which is consistent with the lack of *EPPase* activity observed during insect diapause [20,40]. During insect diapause, embryonic ecdysteroids are mainly found in conjugated form, while subitaneous development has free- and conjugated forms coexisting, which could explain the low expression of *EPPase* [18, 35, 36].

*EcR* exhibited a decrease in expression from 1 to 16 h during subitaneous development, followed by a slight peak at 32 h. This could suggest a more profound peak in *EcR* expression between 16 and 32 h, that would be transcribed prior *ßFTZ-F1* and *E74*, which both peaked at 32 h [9].

Expression of *ßFTZ-F1* gradually decreased from 1 h to 12 h post-oviposition, followed by an increase that statistically peaked at 32 h corresponding to the LB stage. The 1, 4 and 48 h did not result in significant results, which could be due to gradual changes in expression over time. The initial higher levels–and peak the of *ßFTZ-F1* during subitaneous development, probably indicate a previous rise in E20 titer [9].

The initial decrease corresponds to the observed levels of *EPPase*–suggesting that 20E initially are originating from yolk-conjugated ecdysteroids. Since there is no later corresponding a peak in *EPPase* expression, but in *ßFTZ-F1*, this could suggest that the 20E at that point are *de novo* synthesized by Cytochrome P_450_ enzymes encoded by Halloween genes [17,20]. A similar expression pattern was observed for E74, but the decrease after 4 h until the peak at 32 h where, however, abrupt drop rather than a gradually decrease. The matching expression patterns of *ßFTZ-F1* and *E74* corresponds to that *ßFTZ-F1* stimulated the expression of *E74*, which in turn targets CP genes further down the ecdysone-signaling cascade [8,9].

During subitaneous development of the silkworm, *Bombyx mori*, 20E is only present to a minor degree, but around G and O the levels increase rapidly [20]. In *A*. *tonsa*, this could be consistent with the observed peaks in the expression of *ßFTZ-F1* and *E74* that are observed around LB. The observed levels of *ßFTZ-F1* and *E74* are consistent with levels observed in other studies [9].

During quiescence, *EPPase*, *ßFTZ-F1*, and *E74* exhibited a decreasing pattern in expression with lowest levels at 7 d. The expression of *EcR* did not change significantly over time. This suggests that the ecdysone signaling cascade, and thereby ecdysteroids, have a key role in the subitaneous development of *A*. *tonsa*, but not during quiescence. In insects, however, the biological active 20E have been shown to be able to abrupt the dormant state, diapause [41,42]. This suggests that a rise in active ecdysteroids will be able to resume embryogenesis of dormant eggs.

This work provided a detailed description of subitaneous development and quiescence of eggs from our model calanoid species, *A*. *tonsa*. We demonstrated for the first time that embryos would stay in G during quiescence, if the embryos previously had not exceeded that point during subitaneous development. Embryos developed further than G appeared to be present in later stages during quiescence. Hatching from embryos no older than 1 h before induction of quiescence happened at a slower rate in comparison to subitaneous embryos. This suggests that the embryos have to ‘wake up’ from quiescence in G before continuing embryogenesis. Eggs wherein embryos developed to around LB had ‘instant’ hatching within 24 h after the quiescent conditions were terminated, which support the findings of that younger embryos are present in G, while older are present in stages beyond that. The expression patterns of the four genes involved at different levels of the ecdysone-signaling cascade suggest two peaks in 20E titer. The first one at the beginning of embryogenesis, originating from yolk-conjugated ecdysteroids based on the *EPPase* expression. The second around the LB stage probably caused by a peak in *de novo* synthesized 20E, since there are no changes in expression of *EPPase* but of *ßFTZ-F1*, *E74* and possible *EcR*, which are further in the signaling cascade.

Vitiello et al. (2016) found a high egg-mortality of 80% after 150 days of cold-storage for a Mediterranean *A*. *tonsa* strain [43]. The eggs were collected 18-24h after spawning at 20–21°C [43]. Furthermore, it was found that eggs stored from 90 to 240 days required more time to hatch that eggs stored for a shorter period [43]. The higher temperature in Vitiello et al. (2016) the first 18-24h of embryogenesis, as well as strain-differences may explain the significant loss of viable eggs during their egg cold-storage [43]. Strains of *A*. *tonsa* originating from different geographic regions have shown significant differences in terms of cold-storage capacity [44]. Other strains may reach the G stage at different rates.

*A*. *tonsa* and 41 other marine copepod species have been reported to produce resting eggs [3]. Embryos undergoing dormancy constitute ecological and evolutionary reservoirs that are able to recruit new individuals to a pelagic population and ensure its survival during environmental change. Species capable of having egg banks of viable dormant eggs will have a better chance of survival during adverse conditions, in comparison to species without [45].

We suggest that time for initiating embryonic quiescence is of imperative significance for embryonic survival in natural egg banks vital for later re-inoculation of the pelagic after e.g. overwintering [46]. Moreover, it is an aspect of relevance for optimizing protocols for generating culture egg banks in relation to live feed products for marine fish hatcheries [44,47]. The findings of the present study open new opportunities for cold-storing copepod eggs.

## Supporting information

S1 Table Selected developmental times for sampling embryos of the subitaneous development and quiescent state for real-time quantitative PCR and description of the corresponding developmental stages. (PDF)

S2 Table Real-time quantitative PCR primers for the following genes: *ecdysteroid-phosphate phosphatase* (*EPPase*), *ecdysone receptor* (*EcR*), *ß fushi tarazu transcription factor 1* (*ßFTZ-F1*), *ecdysteroid-regulated early gene E74* (*E74*), *ATP synthase* (*ATPS*) and *Histone 3* (*HIST*). Accession numbers, similarity percentages and E-values are given for the species used to search for the gene sequences in the *Acartia tonsa* transcriptome with accession number: GFWY00000000. The sequences were extracted from the transcriptome and the primers generated. F: forward primer, R: reverse primer.
(PDF)

## References

[1] Turner JT. The importance of small pelagic planktonic copepods and their role in pelagic marine food webs. Zool Stud. 2004;43: 255–266.

[2] Paffenhöfer G, Stearns D. Why is *Acartia tonsa* (Copepoda: Calanoida) restricted to nearshore environments? Mar Ecol Prog Ser. 1988;42: 33–38. doi: 10.3354/meps042033

[3] Holm MW, Kiørboe T, Brun P, Licandro P, Almeda R, Hansen BW. Resting eggs in free living marine and estuarine copepods. J Plankton Res. 2018;40: 2–15. doi: 10.1093/plankt/fbx062

[4] Marcus NH. Ecological and evolutionary significance of resting eggs in marine copepods: past, present, and future studies. Hydrobiologia. 1996;320: 141–152. doi: 10.1007/BF00016815

[5] Hairston NG. Temporal dispersal: Ecological and evolutionary aspects of zooplankton egg banks and the role of sediment mixing. Integr Comp Biol. 2002;42: 481–491. doi: 10.1093/icb/42.3.481 21708742 10.1093/icb/42.3.481

[6] Sabatini ME. The developmental stages (copepodids I to Vi) of *Acartia tonsa Dana*, 1849 (Copepoda, Calanoida). Crustaceana. 1990;59: 53–61.

[7] Yang C-M. The egg development of *Paracalanus crassirostris* Dahl, 1894 (copepoda, calanoida). Crustaceana. 1977;33: 33–38.

[8] Subramoniam T. Crustacean ecdysteriods in reproduction and embryogenesis. Comp Biochem Physiol—C Pharmacol Toxicol Endocrinol. 2000;125: 135–156. doi: 10.1016/S0742-8413(99)00098-5 10.1016/s0742-8413(99)00098-5 11790337

[9] Shahin R, Iwanaga M, Kawasaki H. Cuticular protein and transcription factor genes expressed during prepupal-pupal transition and by ecdysone pulse treatment in wing discs of *Bombyx mori*. Insect Mol Biol. 2016;25: 138–152. doi: 10.1111/imb.12207 26748620 10.1111/imb.12207

[10] Glass H, Emmerich H, Spindler K-D. Immunohistochemical localisation of ecdysteroids in the follicular epithelium of locust oocytes. Cell Tissue Res. 1978;194: 237–244. doi: 10.1007/BF00220391 365340 10.1007/BF00220391

[11] Goltzené F, Laguex M, Charlet M, Hoffmann JA. The follicle cell epithelium of maturing ovaries of Locusta migratoria: a new biosynthetic tissue for ecdysone. Hoppe-Seyler´s Zeitschrift für Physiol Chemie. 1978;359: 1427–1434. doi: 10.1515/bchm2.1978.359.2.1427 10.1515/bchm2.1978.359.2.1427 721073

[12] Dinan LN, Rees HH. The identification and titres of conjugated and free ecdysteroids in developing ovaries and newly-laid eggs of *Schistocerca gregaria*. J Insect Physiol. 1981;27: 51–58. doi: 10.1016/0022-1910(81)90032-9

[13] Gande AR, Morgan ED, Wilson ID. Ecdysteroid levels throughout the life cycle of the desert locust, *Schistocerca gregaria*. J Insect Physiol. 1979;25: 669–675. doi: 10.1016/0022-1910(79)90117-3

[14] Lagueux M, Harry P, Hoffmann JA. Ecdysteroids are bound to vitellin in newly laid eggs of locusta. Mol Cell Endocrinol. 1981;24: 325–338. doi: 10.1016/0303-7207(81)90007-1 7035253 10.1016/0303-7207(81)90007-1

[15] Tawfik AI, Vedrová A, Sehnal F. Ecdysteroids during ovarian development and embryogenesis in solitary and gregarious *Schistocerca gregaria*. Arch Insect Biochem Physiol. 1999;41: 134–143. doi: 10.1002/(SICI)1520-6327(1999)41:3<134::AID-ARCH4>3.0.CO;2-6 10398336 10.1002/(SICI)1520-6327(1999)41:3<134::AID-ARCH4>3.0.CO;2-6

[16] Bownes M, Shirras A, Blair M, Collins J, Coulson A. Evidence that insect embryogenesis is regulated by ecdysteroids released from yolk proteins. Proc Natl Acad Sci. 1988;85: 1554–1557. doi: 10.1073/pnas.85.5.1554 3125550 10.1073/pnas.85.5.1554 PMC279811

[17] Niwa R, Niwa YS. Enzymes for ecdysteroid biosynthesis: their biological functions in insects and beyond. Biosci Biotechnol Biochem. 2014;78: 1283–1292. doi: 10.1080/09168451.2014.942250 25130728 10.1080/09168451.2014.942250

[18] Talbot WS, Swyryd E a, Hogness DS. Drosophila tissues with different metamorphic responses to ecdysone express different ecdysone receptor isoforms. Cell. 1993;73: 1323–1337. doi: 10.1016/0092-8674(93)90359-X 8324824 10.1016/0092-8674(93)90359-x

[19] Kozlova T, Thummel CS. Essential rotes for ecdysone signaling during Drosophila mid-embryonic development. Science (80-). 2003;301: 1911–1914. doi: 10.1126/science.1087419 12958367 10.1126/science.1087419

[20] Sonobe H, Yamada R. Ecdysteroids during early embryonic development in silkworm bombyx mori: metabolism and functions. Zoolog Sci. 2004;21: 503–516. doi: 10.2108/zsj.21.503 15170054 10.2108/zsj.21.503

[21] Martini A, Chieco C, Dindo ML, Baronio P. The embryonic development of *Diprion pini* and the related ecdysteroid levels. Bull Insectology. 2011;64: 253–262.

[22] Bordes-Alléaume N, Sami L. Ecdysteroid titres and cuticle depositions in embryos of the dipteran *Calliphora erythrocephala*. Int J Invertebr Reprod Dev. 1987;11: 109–121. doi: 10.1080/01688170.1987.10510270

[23] Yamada M, Murata T, Hirose S, Lavorgna G, Suzuki E, Ueda H. Temporally restricted expression of transcription factor betaFTZ-F1: significance for embryogenesis, molting and metamorphosis in *Drosophila melanogaster*. Development. 2000;127: 5083–92. doi: 10.1128/MCB.16.11.6509 11060234 10.1242/dev.127.23.5083

[24] Støttrup JG, Richardson K, Kirkegaard E, Pihl NJ. The cultivation of *Acartia tonsa* Dana for use as a live food source for marine fish larvae. Aquaculture. 1986;52: 87–96. doi: 10.1016/0044-8486(86)90028-1

[25] Guillard RR, Ryther JH. Studies of marine planktonic diatoms. I. *Cyclotella nana* Hustedt, and *Detonula confervacea* (cleve) Gran. Can J Microbiol. 1962;8: 229–239. doi: 10.1139/m62-029 13902807 10.1139/m62-029

[26] Berggreen U, Hansen B, Kiørboe T. Food size spectra, ingestion and growth of the copepod *Acartia tonsa* during development: implications for determination of copepod production. Mar Biol. 1988;99: 341–352. doi: 10.1007/BF02112126

[27] Thompson PA. Plankton. A Guide to Their Ecology and Monitoring for Water Quality [Internet]. Suthers I.M., Rissik D, editor. Austral Ecology. CSIRO Publishing; 2012 doi: 10.1111/j.1442-9993.2012.02360.x

[28] Zirbel MJ, Miller CB, Batchelder HP. Staging egg development of marine copepods with DAPI and PicoGreen. Limnol Oceanogr Methods. 2007;5: 106–110. doi: 10.4319/lom.2007.5.106

[29] Perkins JR, Dawes JM, McMahon SB, Bennett DL, Orengo C, Kohl M. ReadqPCR and NormqPCR: R packages for the reading, quality checking and normalisation of RT-qPCR quantification cycle (Cq) data. BMC Genomics. 2012;13: 296 doi: 10.1186/1471-2164-13-296 22748112 10.1186/1471-2164-13-296 PMC3443438

[30] Livak KJ, Schmittgen TD. Analysis of relative gene expression data using real-time quantitative PCR and the 2(-Delta Delta C(T)) Method. Methods. 2001;25: 402–8. doi: 10.1006/meth.2001.1262 11846609 10.1006/meth.2001.1262

[31] R T. R Core Team. R: A language and environment for statistical computing R Foundation for Statistical Computing, Vienna, Austria In: https://www.R-project.org/. 2017.

[32] Hansen BW, Drillet G, Kozmér A, Madsen K V., Pedersen MF, Sørensen TF. Temperature effects on copepod egg hatching: does acclimatization matter? J Plankton Res. 2010;32: 305–315. doi: 10.1093/plankt/fbp122

[33] Nilsson B, Jepsen PM, Rewitz K, Hansen BW. Expression of *hsp70* and *ferritin* in embryos of the copepod *Acartia tonsa* (Dana) during transition between subitaneous and quiescent state. J Plankton Res. 2013;36: 513–522. doi: 10.1093/plankt/fbt099

[34] Hansen BW, Drillet G, Pedersen MF, Sjøgreen KP, Vismann B. Do *Acartia tonsa* (Dana) eggs regulate their volume and osmolality as salinity changes? J Comp Physiol B Biochem Syst Environ Physiol. 2012;182: 613–623. doi: 10.1007/s00360-012-0646-y 22270551 10.1007/s00360-012-0646-y

[35] Holmstrup M, Overgaard J, Sorensen TF, Drillet G, Hansen BW, Ramlov H, et al Influence of storage conditions on viability of quiescent copepod eggs (Acartia tonsa Dana): effects of temperature, salinity and anoxia. Aquac Res. 2006;37: 625–631. doi: 10.1111/j.1365-2109.2006.01472.x

[36] Drillet G, Iversen MH, Sørensen TF, Ramløv H, Lund T, Hansen BW. Effect of cold storage upon eggs of a calanoid copepod, *Acartia tonsa* (Dana) and their offspring. Aquaculture. 2006;254: 714–729. doi: 10.1016/j.aquaculture.2005.11.018

[37] Drillet G, Jørgensen NOG, Sørensen TF, Ramløv H, Hansen BW. Biochemical and technical observations supporting the use of copepods as live feed organisms in marine larviculture. Aquac Res. 2006;37: 756–772. doi: 10.1111/j.1365-2109.2006.01489.x

[38] Støttrup JG, Jensen J. Influence of algal diet on feeding and egg-production of the calanoid copepod *Acartia tonsa* Dana. J Exp Mar Bio Ecol. 1990;141: 87–105. doi: 10.1016/0022-0981(90)90216-Y

[39] Chen F, Marcus NH. Subitaneous, diapause, and delayed-hatching eggs of planktonic copepods from the northern Gulf of Mexico: morphology and hatching success. Mar Biol. 1997;127: 587–597. doi: 10.1007/s002270050049

[40] Asada M, Kato Y, Matsuura T, Watanabe H. Early embryonic expression of a putative Ecdysteroid-Phosphate Phosphatase in the water flea, Daphnia magna (Cladocera: Daphniidae). J Insect Sci. 2014;14: 1–6. doi: 10.1093/jis/14.1.1 25399434 10.1093/jisesa/ieu043 PMC5634057

[41] Makka T, Seino A, Tomita S, Fujiwara H, Sonobe H. A possible pole of 20-hydroxyecdysone in embryonic development of the silkworm *Bombyx mori*. Arch Insect Biochem Physiol. 2002;51: 111–120. doi: 10.1002/arch.10055 12386839 10.1002/arch.10055

[42] Gharib B, Girardie A, De Reggi M. Ecdysteroids and control of embryonic diapause: changes in ecdysteroid levels and exogenous hormone effects in the eggs of *cochineal Lepidosaphes*. Experientia. 1981;37: 1107–1108.

[43] Vitiello V, Zhou C, Scuderi A, Pellegrini D, Buttino I. Cold storage of *Acartia tonsa* eggs: a practical use in ecotoxicological studies. Ecotoxicology. Springer US; 2016;25: 1033–1039. doi: 10.1007/s10646-016-1660-8 27106013 10.1007/s10646-016-1660-8

[44] Hansen BW, Buttino I, Cunha ME, Drillet G. Embryonic cold storage capability from seven strains of *Acartia* spp. isolated in different geographical areas. Aquaculture. Elsevier B.V.; 2016;457: 131–139. doi: 10.1016/j.aquaculture.2016.02.024

[45] Hairston NG. Zooplankton egg banks as biotic reservoirs in changing environments. Limnol Oceanogr. 1996;41: 1087–1092. doi: 10.4319/lo.1996.41.5.1087

[46] Sichlau MH, Hansen JLS, Andersen TJ, Hansen BW. Distribution and mortality of diapause eggs from calanoid copepods in relation to sedimentation regimes. Mar Biol. 2011;158: 665–676. doi: 10.1007/s00227-010-1590-6

[47] Hansen BW, Blanda E, Drillet G, Højgaard JK, Mahjoub M-S, Rayner TA. Outdoor rearing facilities of free spawning calanoid copepods for turbot larva can host a bank of resting eggs in the sediment. Aquac Int. Springer International Publishing; 2016;24: 949–964. doi: 10.1007/s10499-015-9963-y

