# Supplementary material for: Timing of embryonic quiescence determines viability of embryos from the calanoid copepod, Acartia tonsa (Dana)
Source: PLoS One. 2018 Mar 7;13(3):e0193727. doi: 10.1371/journal.pone.0193727 (PMC5841787; doi:10.1371/journal.pone.0193727)
Supplement: S1 Table — (PDF) [file pone.0193727.s001.pdf]

S1 Table. Selected developmental times for sampling embryos of the subitaneous development and quiescent state for real-time quantitative PCR and description of the corresponding developmental stages.

Time	Subitanous development	Quiescent state
**1 h**	Majority of embryos in a single- or two celled stage (S1, S2)	Majority of embryos in a single- or two celled stage (S1, S2)
**4 h**	Majority of embryos in a 64 (S7), 128 – or more celled stage (S8).	S3 – S4
**5 h**	Majority of embryos beginning gastrulation (G)	Embryos ranging from a 16- to a 64-celled stage (S5, S6, S7).
**7 h**	Majority of embryos in gastrulation (G)	16-celled stage (7S)
**12 h**	Beginning organogenesis (O)	128-celled stage – or more (S8)
**16 h**	Majority in organogenesis (O)	128-celled stage – or more (S8)
**32 h**	Majority in limb bud stage, LB	128-celled stage – or more (S8)
**48 h**	Early or final nauplii, EN or FN	Gastrulation (G)
**7d**		Gastrulation (G)
