# Supplementary material for: Timing of embryonic quiescence determines viability of embryos from the calanoid copepod, Acartia tonsa (Dana)
Source: PLoS One. 2018 Mar 7;13(3):e0193727. doi: 10.1371/journal.pone.0193727 (PMC5841787; doi:10.1371/journal.pone.0193727)
Supplement: S2 Table — Accession numbers, similarity percentages and E-values are given for the species used to search for the gene sequences in the Acartia tonsa transcriptome with accession number: GFWY00000000. The sequences were extracted from the transcriptome and the primers generated. F: forward primer, R: reverse primer. (PDF) [file pone.0193727.s002.pdf]

S2 Table. Real-time quantitative PCR primers for the following genes: *ecdysteroid-phosphate phosphatase* (*EPPase*), *ecdysone receptor* (*EcR*), *ß fushi tarazu transcription factor 1* (*ßFTZ-F1*), *ecdysteroid-regulated early gene E74* (*E74*), *ATP synthase* (*ATPS*) and *Histone 3* (*HIST*). Accession numbers, similarity percentages and E-values are given for the species used to search for the gene sequences in the *Acartia tonsa* transcriptome with accession number: GFWY00000000. The sequences were extracted from the transcriptome and the primers generated. F: forward primer, R: reverse primer.

Gene	Primers	Amplicon	Accession	Similarity	E-value
EPPase	**F:** CATGGAGAGAGGGTAGACTTTACT<br>**R:** AAATCTTTTCTTTCATAGTTTCCAGCA	83 bp	AB742158<br>*Daphnia magna*	72.8 %	1.69e-05
EcR	**F:** CGGCCACAGGAGGAATTTTG<br>**R:** CGGAAAAATCCCTTGCACCC	95 bp	JX105044<br>*Bactrocera dorsalis*	68.3%	1.62e-24
ßFTZ-1	**F:** CACTCCGACACAAAGGAAGGA<br>**R:** GACTCGCAGGTTAGCAGGC	92 bp	LC105701 *Daphnia magna*	79.4%	6.19e-19
E74	**F:** GTACCGGGCCTGTATCTGTG<br>**R:** GAGGAAAGGCTCCCCGTAAG	95 bp	NM001043979<br>*Bombyx mori*	75.1%	2.31e-44
ATPS	**F:** TCCAGAAGGAACGCCGGC<br>**R:** AGCACCTAGACGAACAGCTA	100 bp	NC014887.1<br>*Acrida cinerea*	69.1%	1.22e-24
HIST	**F:** GTACCGGGCCTGTATCTGTG<br>**R**: GAGGAAAGGCTCCCCGTAAG	100 bp	NW001887649.1<br>*Culex quinquefasciatus*	79.5%	1.16e-99
